# Complete mitochondrial genome of the deep-sea asymmetrical barnacle *Altiverruca navicula* (Cirripedia, Thoracica, Verrucumorpha)

**DOI:** 10.1080/23802359.2017.1413297

**Published:** 2017-12-08

**Authors:** Se-Joo Kim, Won-Kyung Lee, Bo Kyeng Hou, Benny K. K. Chan, Se-Jong Ju

**Affiliations:** a Korean Bioinformation Center, Korea Research Institute Bioscience and Biotechnology, Daejeon, South Korea;; b Deep-Sea and Seabed Mineral Resources Research Center, Korea Institute of Ocean Science & Technology, Ansan, South Korea;; c Biodiversity Research Center, Academica Sinica, Taipei, Taiwan;; d Marine Biology Major, University of Science & Technology, Daejeon, South Korea

**Keywords:** *Altiverruca navicula*, barnacle, Fiji Basin, mitochondrial genome, Verrucomorpha

## Abstract

The hitherto suborder Verrucomorpha contains asymmetrical barnacles of two groups: the true Verrucomorpha (*Eoverruca* + Verrucidae) and the Neoverrucidae. Here, we determined the mitochondrial genome (mitogenome) of *Altiverruca navicula*, a true Verrucomorpha species. The mitogenome was 15,976 base pairs in length and had the typical pancrustacean gene arrangement. Its protein-coding genes were very similar to those of other thoracican species in terms of length, AT content, and start and stop codons. In phylogenetic trees constructed with 13 protein-coding genes, *A*. *navicula* was positioned at an ancestral node of sessile barnacles, consistent with the findings of previous studies.

The suborder Verrucomorpha, recognized as basal sessilians, is asymmetrical barnacles which are characterized primarily by the status of their paired scuta and terga (movable or fixed). Based on morphological and molecular studies, its members have been divided into two groups, the true Verrucomorpha (fossil *Eoverruca* + Verrucidae) and the Neoverrucidae, which is restricted to hydrothermal vent habitats and has a challenge to revise its taxonomic status (Pérez-Losada et al. 2014; Gale 2015; Herrera et al. 2015). Among the true Verrucomorpha, the genus *Altiverruca* is one of the most common deep-sea barnacles (Buckeridge 1994; Young 2002). Although several previous studies have investigated the evolution of verrucomorphs, little is known about their phylogenetic status at a genomic level. To understand verrucomorphs, we determined the mitochondrial genome (mitogenome) of *Altiverruca navicula* (Hoek 1913).

In December 2016, *A*. *navicula* specimens were collected from the North Fiji Basin (17°6′S 173°52′W; 2255 m in depth) in the southwestern Pacific Ocean using a ROV (ROPOS, Canadian Scientific Submersible Facility). Genomic DNA was extracted using the QIAamp Fast DNA Tissue Kit (QIAGEN, Hilden, Germany) and mitochondrial DNA was amplified with a DNA REPLI-g Mitochondrial DNA Kit (QIAGEN, Hilden, Germany). Library construction and sequencing were performed by Macrogen Service (Macrogen, Seoul, Korea) using the Illumina HiSeq 4000 sequencing platform (Illumina, San Diego, CA). A complete mitochondrial genome was obtained using NOVOplasty 2.4 (Dierckxsens et al. 2017) and Geneious (Biomatters, Auckland, New Zealand). Mitochondrial genes were annotated using MITOS (Bernt et al. 2013) and ARWEN (Laslett and Canbäck 2008). The used specimen for mitogenome analysis had been deposited in the Library of Marine Samples (KIOST, Geojae, Korea; accession no. BS_MA00010132).

The complete mitogenome of *A*. *navicula* was 15,976 bp in length (GenBank accession no. MG252956), and consisted of 13 protein-coding genes (PCGs), two ribosomal RNAs (rRNAs), 22 transfer RNAs (tRNAs) and a non-coding region. The gene organization largely followed the ancestral pancrustacean pattern, except for some tRNAs. The base composition was 38.2% A, 17.0% C, 10.4% G and 34.4% T. All PCGs had an ATN start codon, except COX1, which was initiated with TTG. Most of the PCGs terminated with a complete stop codon (TAA or TAG), but four PCGs (COX1, COX3, ND3 and ND4) had incomplete stop codons (T−). The 16 and 12S rRNAs were 1295 bp (77.5% AT content) and 755 bp (72.3% AT content), respectively. The tRNA genes ranged from 61 to 70 bp in size. A 443 bp-long (79.7% AT content) non-coding region was located between the 12S rRNA and tRNA*^Lys^*.

Phylogenetic trees were constructed with the PCGs of 13 barnacles using maximum-likelihood (ML) and Bayesian inference (Figure 1). Although ML and Bayesian trees showed different relationships among balanomorphs, both methods positioned *A*. *navicula* at an ancestral node of sessile barnacles, which concurs with the previous studies (Pérez-Losada et al. 2014; Gale 2015; Herrera et al. 2015). Considering the biodiversity and the taxonomic ambiguity of verrucomorphs, further mitogenomic analysis of undetermined taxa is required. Our results will contribute to the understanding of barnacle phylogeny, evolution and biogeography.

**Figure 1.**
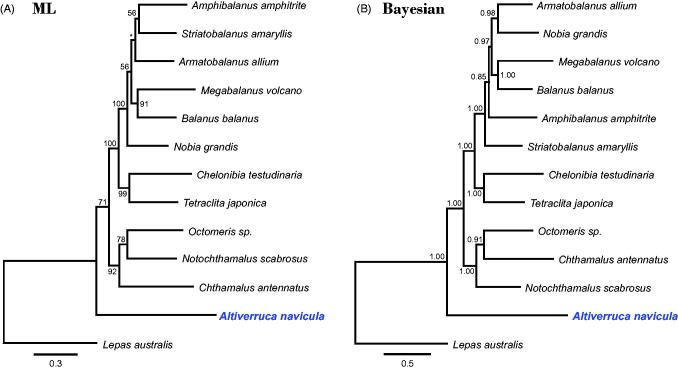
Phylogenetic tree of *Altiverruca navicula* and related barnacles based on 13 protein-coding genes from mitogenomes. The model GTR + I + G was selected as the best evolutionary model using jModelTest 2.1.4. Numbers on internodes are the maximum-likelihood bootstrap proportions (A) and Bayesian posterior probabilities (B). An asterisk indicates a bootstrap value of less than 50%. The accession numbers of barnacles are as follows: *Altiverruca navicula*, MG252956; *Amphibalanus amphitrite*, NC_024525; *Armatobalanus allium*, NC_029167; *Balanus balanus*, NC_026466; *Chelonibia testudinaria*, NC_029169; *Chthamalus antennatus*, NC_026730; *Lepas australis*, NC_025295; *Megabalanus volcano*, NC_006293; *Nobia grandis*, NC_023945; *Notochthamalus scabrosus*, NC_022716; *Octomeris* sp., KJ754820; *Striatobalanus amaryllis*, NC_024526; and *Tetraclita japonica*, NC_008974.
